# Psychometric Properties and Factor Structure of the Chinese Version of the Hospital Anxiety and Depression Scale in People Living With HIV

**DOI:** 10.3389/fpsyt.2019.00346

**Published:** 2019-05-16

**Authors:** Zhiyuan Yang, Xiaojie Huang, Xinchao Liu, Jianhua Hou, Wenfang Wu, Aixin Song, Kathrine Meyers, Tong Zhang, Hui Chen, Hao Wu

**Affiliations:** ^1^Peking University People’s Hospital, Peking University Health Science Center, Beijing, China; ^2^Center for Infectious Diseases, Beijing You’an Hospital, Capital Medical University, Beijing, China; ^3^Department of Infectious Diseases, Peking Union Medical College Hospital, Beijing, China; ^4^School of Biomedical Engineering, Capital Medical University, Beijing, China; ^5^The Aaron Diamond AIDS Research Center, New York, NY, United States

**Keywords:** Hospital Anxiety and Depression Scale, internal reliability, factor analysis, acquired immune deficiency syndrome, human immunodeficiency virus

## Abstract

The population of people living with HIV (PLWH) is growing in number and usually results in mental health problems that impact their quality of life. Therefore, valid instruments and screening methods for psychological disorders are of great significance. The Hospital Anxiety and Depression Scale (HADS) reveals good psychometric properties, but shows ambiguous results in factor structure. This study aims to evaluate psychometric properties in terms of the internal reliability and structure validity of the Chinese version of the HADS (C-HADS) in a large sample of PLWH in China. The C-HADS was administered to 4,102 HIV-infected adults at an HIV clinic in China. Exploratory factor analysis (EFA) and confirmatory factor analysis (CFA) were performed to examine the factor structure. Measurement invariance was assessed across gender and course of infection. Internal reliability was also assessed. A bifactor model with anomalous loadings of items 7, 8, and 10 fits the data best and holds measurement invariance across gender and course of infection. Internal reliability was good with all Cronbach’s alphas > 0.70 and Spearman’s ρ between 0.30 and 0.70. The C-HADS has good psychometric properties in terms of internal reliability and structure validity of a bifactor model. The C-HADS is recommended to be used as a total scale that measures general psychological distress, instead of anxiety and depression separately, when applied to PLWH. Further studies are needed to evaluate criterion validity, the cutoff score, and the effect of wording and scoring of the HADS.

## Introduction

As reported by the World Health Organization (WHO), HIV has been a constant major public health concern worldwide during the past few decades. The number of people living with HIV (PLWH) was reported to be around 36.7 million at the end of 2016, with 1.8 million newly infected cases in 2016 globally (1). Newly diagnosed cases in China have soared in the past few years. HIV/AIDS is considered a life-threatening and chronic disease that leads to various mental health problems among PLWH. A systematic review and meta-analysis showed that the prevalence of depression and/or anxiety in PLWH was about one-third, higher than for patients living with other chronic medical conditions and the general population (2). A recent systematic review in China revealed a higher prevalence of depression (greater than 60%) and anxiety (greater than 40%) in a sample of PLWH (3), which is associated with higher odds of HIV-related outcomes, such as poor adherence to antiviral therapy (ART), suboptimal engagement with HIV services, and worse HIV clinical outcomes (4, 5). While mental health in PLWH deserves more attention, this urgent issue has been largely ignored in policy guidelines globally (6). Thus, it is critical for clinicians to develop a reliable and valid instrument to screen for mental disorders among this population.

The Hospital Anxiety and Depression Scale (HADS) (7) is a 14-item self-report scale consisting of two subscales, anxiety and depression. The HADS is not only relatively brief but also more suitable for patients with comorbid physical problems, including PLWH, as it excludes somatic items. Moreover, the HADS scores were found to be unconfounded by the presence of HIV symptomatology (8). The HADS’s psychometric properties have been tested since its proposal. A systematic review and meta-analysis including 747 studies suggested good internal reliability. Cronbach’s α for the anxiety subscale ranged from 0.68 to 0.93 (mean 0.83) and that for the depression subscale ranged from 0.67 to 0.90 (mean 0.82) and had good or better concurrent validity (correlations between the HADS and other commonly used questionnaires varied from 0.49 to 0.83) and sensitivity and specificity (approximately 0.80) with a cutoff score of 7/8 (9). The aforementioned findings were further supported by more recent studies, indicating good internal reliability (10–15), good or strong correlations (11, 13), and acceptable sensitivity and specificity (16, 17).

Although the HADS is considered robust across a number of psychometric properties, its factor structure has long been debated. The original factor model proposed by Zigmond and Snaith (7) was a two-factor model with correlation between anxiety and depression that was supported by an early review (9). Meanwhile, other models also received support, such as Dunbar’s high-order model (18), which was developed from the tripartite theory of anxiety and depression. Inconsistency remained and led to an appeal to abandon the HADS (19). Norton and colleagues (20) point out that an overarching general distress factor may be the explanation for the inconsistent results coming from studies of the structure of the HADS. Based on an investigation of the symptoms of anxiety and depression, they proposed a bifactor model (20), containing one general factor and several group factors, which was supported by a meta-CFA study (20). The bifactor model consists of a general factor, namely, general distress, which is a broad factor that includes all observed items, and conceptually narrower group factors, such as anxiety and depression, consisting of observed items with related content. Subsequently, it became a trend to test bifactor models in different samples, and bifactor models were proven to be the best solution among ischemic heart disease patients (21), community populations (22), and Huntington’s disease patients (23). The group factors from those studies vary, which could contain the same items as the original (21, 22) or proposed subscales (20), or they were extracted through an exploratory factor analysis (EFA) study (23, 24). Apart from the classic test theories (CTTs) characterized by EFA, modern test theories, such as item response theory (IRT), have been applied to test the factor structure of the HADS (25). Different from the classical test theory, IRT explores the interaction between item and the person, evaluates the person’s ability and item difficulty separately, and is able to assess the difference in item interpretation between different groups of people. Some studies have applied both CTT and IRT models to the factor analysis of the HADS (14, 24).

The factor structure of the HADS was also tested in PLWH, resulting in a two-factor model (8) and a unidimensional model (26). Inconsistency also exists across studies using the Chinese version of HADS, which used a two-factor model (27), a modified two-factor model (28, 29, 30), a three-factor model (31), and a bifactor model (24). The incongruity may be due to the heterogeneity regarding the locations of the studies and the patients recruited, indicating the necessity for this study. Moreover, there are special subgroups in the population of PLWH that require more attention. Systematic reviews suggested that the prevalence of depression and anxiety in women is greater than that in men (2, 3). Men who have sex with men (MSM) have a relatively higher tendency toward negative perceptions of HIV/AIDS both cognitively and emotionally (32) and demonstrate a higher prevalence of anxiety and depression when compared to other patients (33). As the prevalence is considerably higher in those special subgroups, there is a need to test if the HADS is an invariant measurement for patients of different genders or course of infection. This study aimed to present an evaluation of the psychometric properties of the Chinese version of HADS (C-HADS) in terms of internal reliability and structure validity in a large sample of PLWH in China.

## Methods

### Procedures and Participants

The current study is a cross-sectional study conducted in 20 HIV treatment clinics across China, including Beijing, Yunnan, Guangxi, and other major provinces and municipalities. Those 20 clinics were selected for the following reasons: 1) designated by the government for antiretroviral treatment; 2) Grade III Level A; and 3) had a considerable amount of typical antiviral-treatment cases, which is consistent with previous articles based on the same dataset. Participants in the study had to meet the following criteria: 1) at least 18 years of age; 2) diagnosed with an HIV infection; 3) on antiretroviral therapy; and 4) not pregnant within the prior 3 months. Participants should not simultaneously have any medical condition that could impede their ability to complete the questionnaire of the current study. All participants signed the written informed consent and were provided with the questionnaire, which consisted of the Chinese version of the HADS and basic information, such as gender, age, and sexual orientation. Of the 4,724 questionnaires collected, 4,102 (86.83%) were usable, while others were excluded because they were incomplete. The study was approved by Beijing You’an Hospital’s institutional review board.

### Measures

The HADS is a commonly used self-report scale, consisting of a seven-item anxiety subscale (HADS-A) and a seven-item depression subscale (HADS-D). Item responses are graded on a four-point Likert-type scale (0–3), indicating the severity of each symptom during the prior week. The score is summed up separately with a cutoff score of 8 in each subscale (7).

### Data Analysis

Demographic characteristics and descriptive statistics (frequencies with proportions for qualitative data and means ± standard deviations for quantitative data) were presented, and the differences in the HADS-A and the HADS-D scores between the groups of patients were tested with independent samples *t* tests.

The total sample of 4,102 patients was randomly split into two subsamples (*n* = 2,051 in each) to conduct EFA or CFA, respectively. The randomness of the split was guaranteed by using Microsoft Excel-generated random numbers to place patients into a random order and divide them into half. For EFA, a principal component analysis (PCA) with Varimax rotation was conducted with the first subsample to explore the underlying factor structure of the HADS. Meaningful loadings were evaluated by a criterion of 0.32 (poor), 0.45 (fair), 0.55 (good), 0.63 (very good), or 0.71 (excellent) (34).

A confirmatory factor analysis (CFA) was performed with the second subsample to evaluate whether a formerly proposed model or the results from the EFA could explain the HADS’ structure. The maximum likelihood method, based on Pearson correlation matrix, was used to estimate the parameters of the model. Four models were tested in this study: i) model 1, original model with two correlated factors (7); ii) model 2, a bifactor model, consisting of two unrelated group factors and a general factor (20); iii) model 3, revised model based on EFA with two correlated factors; and iv) model 4, a bifactor model of model 3.

The goodness of fit (GoF) was assessed using the following criteria (35): 1) relative chi-square (c^2^/degree of freedom): <3 suggests an acceptable fit, and <2 suggests a good fit; 2) root mean square error of approximation (RMSEA): <0.06 suggests a good fit, <0.08 suggests an adequate fit, and >0.08 suggests a poor fit; 3) standardized root mean residual (SRMR): <0.06 suggests a good fit, <0.08 suggests an adequate fit, and >0.08 suggests a poor fit); 4) comparative fit index (CFI): >0.95 suggests a good fit, >0.9 suggests an adequate fit, and <0.9 suggests a poor fit; and 5) the Tucker–Lewis index (TLI): the same as the CFI. Parsimony indices were also used (34): 1) parsimony goodness of fit index (PGFI): >0.50 suggests an acceptable, larger value (closer to 1.00) and indicates a better fit and 2) Akaike information criterion (AIC): a CFI without a cutoff, wherein smaller values indicate a better fit.

Measurement invariance was tested with the second subsample to assess whether the HADS could be validly used across HIV patients with different genders and transmission routes. Constrained models (configural, metric, strong, and strict) were assessed with GoF indices under the above criteria. A change of ≤−0.010 for CFI (i.e., ΔCFI ≤ −0.010) and of ≥0.015 for RMSEA (i.e., ΔRMSEA ≥ 0.015) suggests a decrease in model fit and lack of measurement invariance across subgroups (36). Δc^2^ (Δ*p* < 0.05) also indicates a lack of measurement invariance, but it is likely to reject a model with a large sample size, while CFI and RMSEA are slightly dependent on sample size (36). A strong invariance is generally considered adequate for measurement invariance in clinical practice (37).

Cronbach’s α, which is conducted within a scale, and, as the scoring of the subscales deviated from a normal distribution, Spearman’s ρ, which tests correlation between subscales, were calculated to evaluate the internal reliability. Cronbach’s α values > 0.70 (38) and Spearman’s ρ between 0.30 and 0.70 (39) indicate a good internal reliability.

The descriptive statistics, EFA, and internal reliability were analyzed with SPSS 17.0, and CFA and measurement invariance were analyzed with Amos 24.0.

## Results

### Descriptive Statistics

A sample of 4,102 HIV-infected patients were qualified for data analysis with a mean age of 37.6 years (SD: 11.7 years); 79.4% of the patients were male, and 38.5% of the patients were infected through anal sex. The prevalence of anxiety (HADS-A score ≥ 8) and depression (HADS-D score ≥ 8) was 27.4% and 32.9%, respectively. Female patients scored significantly higher than male patients in HADS-A (*p* = 0.001) and HADS-D (*p* < 0.001). MSM scored significantly higher than heterosexual male patients in HADS-A (*p* = 0.027), but lower in HADS-D (*p* < 0.001; **Table 1**).

**Table 1:** Scores of the HADS across patient subgroups.

		Anxiety (*M* ± SD)	*p* value	Depression (*M* ± SD)	*p* value
Gender	Male (*n* = 3,204)	5.20 ± 3.93	0.001	5.55 ± 3.95	<0.001
	Female (*n* = 830)	5.73 ± 4.15		6.22 ± 4.13	
Sexual Orientation	MSM (*n* = 1,580)	5.36 ± 3.79	0.027	5.26 ± 3.83	<0.001
	Heterosexual male (*n* = 1,624)	5.05 ± 4.05		5.83 ± 4.05	

HADS, Hospital Anxiety and Depression Scale.

### Factor Structure

#### Exploratory Factor Analysis

The total sample of 4,102 was randomly split into two subsamples (*n* = 2,051 in each) to conduct EFA or CFA, respectively. With the subsample size of 2,051 and the 14 HADS items, the ratio of participants to items was >146:1, which meets the 10:1 requirement. The Kaiser–Meyer–Olkin (KMO) test indicated that the participant-to-item ratio was sufficient for an EFA test (KMO = 0.910), and a Bartlett’s test also suggested that the sample size was appropriate [c^2^(91) = 7863.573, *p* < 0.001]. Two factors with an eigenvalue > 1 were sought, using a Varimax rotation, accounting for 35.2% and 10.6% of the variance, respectively (**Table 2**). Item loadings that could be considered as fair or better are bolded in **Table 2**.

**Table 2:** Results for the exploratory factor analysis of the HADS.

Item	Factor 1	Factor 2
1	**0.721**	0.114
2	0.084	**0.629**
3	**0.743**	0.136
4	0.040	**0.756**
5	**0.592**	0.357
6	0.438	**0.559**
7	0.309	**0.607**
8	**0.608**	0.099
9	**0.532**	0.280
10	**0.526**	0.187
11	**0.620**	0.139
12	0.255	**0.614**
13	**0.766**	0.149
14	0.128	**0.601**
Eigenvalue	4.928	1.481
Cumulative explained variance (%)	35.200	10.576

Figures in the table were item loadings with item loadings ≥ 0.45 in bold.

Loadings of items 9 and 10 included in factor 1 were fair, and loadings of other items were good or better in their respective factors. No substantial cross-loadings were found. The first factor (factor 1) is composed of eight items, items 1, 3, 5, 8, 9, 10, 11, and 13, and the second factor (factor 2) is composed of six items, items 2, 4, 6, 7, 12, and 14. Factor 1 consists mostly of anxiety items, while factor 2 contains most of the depression items. However, three items were found to have anomalous loadings different from Zigmond and Snaith’s model; item 7, loaded on factor 2 with good loading, and items 8 and 10, loaded onto factor 1 with good and fair loadings, respectively.

#### Confirmatory Factor Analysis

**Figure 1:**
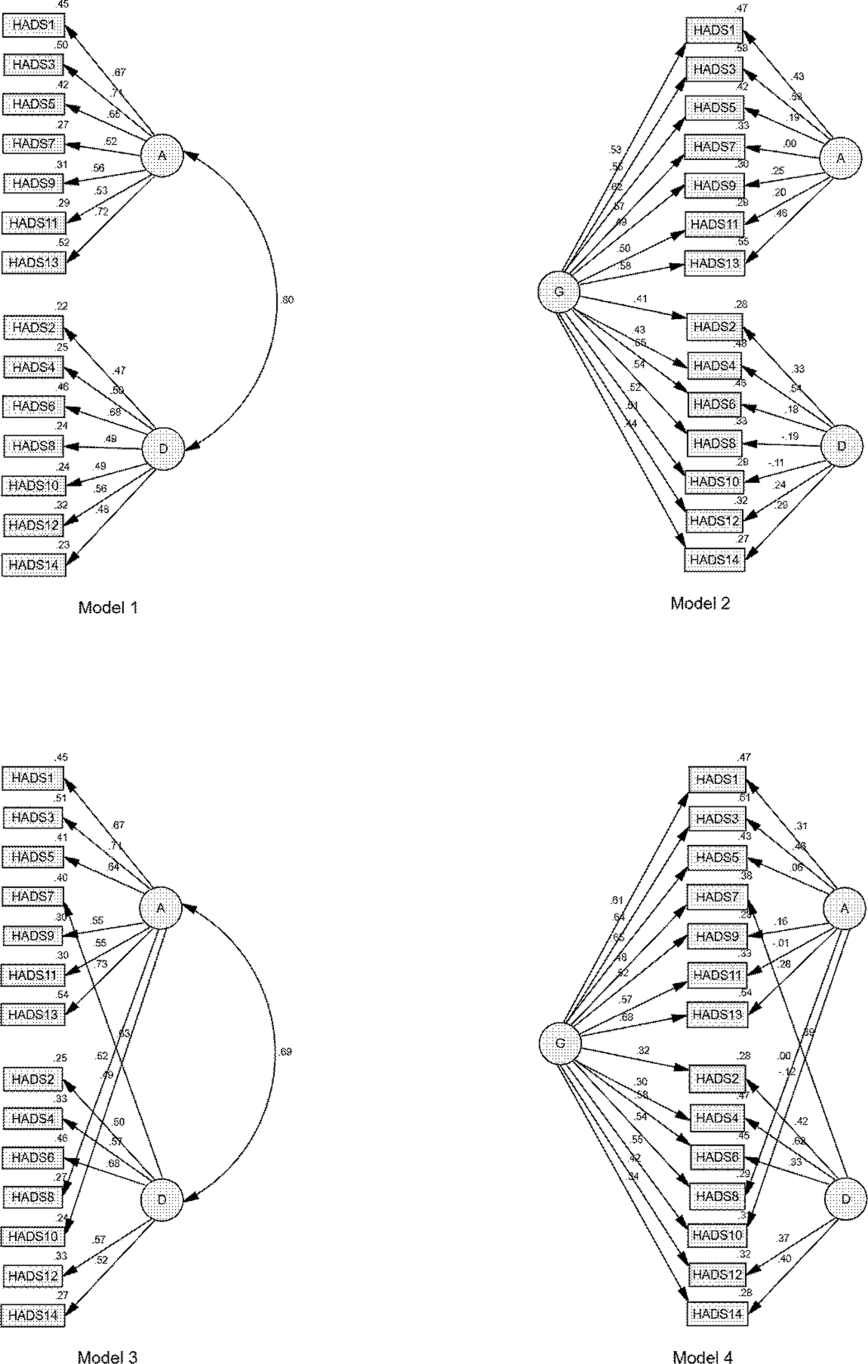
Factor loadings for each tested model. A, anxiety; D, depression; G, general distress. Large ovals represent latent variables; straight lines with arrows represent hypothesized direct effects; rectangles represent measured variables; small ovals represent error terms; and numbers represent standardized parameter estimates.

Four possible models (**Figure 1**) were tested for GoF. The GoF indices for the four tested models are listed in **Table 3**. All models, except the original two-factor model (model 1), could be considered as having an adequate fit. The addition of a general factor (models 2 and 4) improved the fit of the respective two-factor models (models 1 and 3). Model 4 was the only model that met the GoF criteria of a good fit in terms of RMSEA (<0.050) and CFI (>0.950). Model 4 also had the smallest AIC among all the models and an acceptable PGFI (>0.50), although not the largest. These data suggested that model 4, the bifactor model with item 7 being loaded onto HADS-D and items 8 and 10 onto HADS-A, was the best fit for these data.

**Table 3:** Goodness-of-fit indices of different models in the confirmatory factor analysis.

	χ^2^	df	*p*	RMSEA	(90% CI)	SRMR	CFI	TLI	AIC	PGFI
Model 1	1067.495	76	0.000	0.080	0.076 – 0.084	0.064	0.874	0.849	1125.495	0.664
Model 2	458.562	63	0.000	0.055	0.051 – 0.060	0.034	0.950	0.927	542.562	0.581
Model 3	625.360	76	0.000	0.059	0.055 – 0.064	0.046	0.930	0.916	683.360	0.692
Model 4	290.351	63	0.000	0.042	0.037 – 0.047	0.029	0.971	0.958	402.351	0.588

CI, confidence interval; *χ* ^2^, chi-square; df, degrees of freedom; RMSEA, root mean square error of approximation; SRMR, standardized root mean residual; CFI, comparative fit index; TLI, Tucker–Lewis index; AIC, Akaike information criterion; PGFI, Parsimony Goodness-of-fit Index.

### Measurement Invariance

The model that showed the best fit in CFA study (model 4) was first assessed within each subgroup (male and female, and MSM and non-MSM) separately and showed a good fit for all four patient subgroups across all indices (RMSEA, SRMR, TLI, CFI, AIC, and PGFI). The increasingly constrained models (configural, metric, strong, and strict) were then tested (**Table 4**). For groups that were different in gender, RMSEA, SRMR, TLI, CFI, and PGFI of the constrained models met with the adequate criterion with an ΔCFI always >−0.010 and an ΔRMSEA always <0.015, indicating that measurement invariance of the HADS was adequate between genders. For groups different in courses of infection, GoF indices of the constrained models also met with the criterion of strong invariance, but failed in strict invariance where ΔCFI = 0.011. Considering that strong invariance is adequate for measurement invariance in clinical practice, the measurement model works similarly across different courses of infection.

**Table 4:** Measurement invariance test for Model 4.

		χ^2^	(Δχ^2^)	df	(Δdf)	*p*	(Δ*p*)	CFI	(ΔCFI)	RMSEA	(ΔRMSEA)	SRMR	TLI	AIC	PGFI
Gender	Male	268.531		63		0.000		0.967		0.045		0.031	0.952	352.531	0.586
	Female	69.332		63		0.273		0.996		0.016		0.033	0.994	153.332	0.585
	Configural	335.891		126		0.000		0.973		0.029		0.031	0.960	503.891	0.586
	Metric	362.584	26.693	151	25	0.000	0.371	0.972	−0.001	0.026	−0.003				
	Strong	364.042	1.459	154	3	0.000	0.692	0.973	0.001	0.026	0.000				
	Strict	385.416	21.373	168	14	0.000	0.092	0.972	−0.001	0.025	−0.001				
Course of infection	Non-MSM	199.483		63		0.000		0.971		0.042		0.029	0.958	283.483	0.586
	MSM	147.799		63		0.000		0.974		0.041		0.032	0.962	231.799	0.585
	Configural	347.286		126		0.000		0.972		0.029		0.029	0.959	515.286	0.586
	Metric	374.469	27.183	151	25	0.000	0.347	0.972	0.000	0.027	−0.002				
	Strong	379.041	4.572	154	3	0.000	0.206	0.971	−0.001	0.027	0.000				
	Strict	485.228	106.197	168	14	0.000	0.000	0.960	−0.011	0.030	0.003				

*χ* ^2^, chi-square; df, degrees of freedom; RMSEA, root mean square error of approximation; SRMR, standardized root mean residual; CFI, comparative fit index; TLI, Tucker–Lewis index; AIC, Akaike information criterion; PGFI, Parsimony Goodness-of-fit Index.

### Internal Reliability

Cronbach’s α of the total scale was higher than the subscales, with all the α values > 0.70, indicating good internal reliability (**Table 5**). The modified subscales demonstrated better internal reliability than the original subscales. Spearman’s ρ between the subscales were considered moderate (0.628 for the original two-factor model and 0.542 for the modified two-factor model).

**Table 5:** Internal reliability of the HADS subscales and total scale.

	Cronbach’s α	Spearman’s ρ
HADS-A (7 items)	0.811	0.628
HADS-D (7 items)	0.729	
Modified HADS-A (8 items)	0.823	0.542
Modified HADS-D (6 items)	0.755	
HADS-T (14 items)	0.852	

Modified HADS-A (eight items) consists items 1, 3, 5, 8, 9, 10, 11, and 13; Modified HADS-D (six items) consists of items 2, 4, 6, 7, 12, and 14.

## Discussion

In the current study, the psychometric properties of the Chinese version of the HADS have been assessed in a large sample of PLWH. In general, the HADS has demonstrated its validity as an instrument for measuring psychological distress in this sample. We found a bifactor model with anomalous loadings of items 7, 8, and 10; measurement invariance was consistent between genders and courses of infection, and internal consistency was satisfactory.

The current study of 4,012 samples is one of the largest studies ever done that shows the prevalence of anxiety and depression among PLWH in the ART era (7, 40, 41, 42). The prevalence of anxiety and depression in the sample of 4,102 PLWH in the current study was 27.4% and 32.9%, respectively, which is consistent with the data of low- and middle-income countries in a worldwide systematic review (anxiety—mean: 33.92%, SD: 10.64%; depression—mean: 41.36%, SD: 21.42%) (2), but it was slightly lower than a systematic review in China [anxiety: 11.11%–97.53% (median prevalence: 43.1%), depression: 16%–100% (median prevalence: 60.4%)] (3). However, many former studies conducted in China analyzed PLWH that were infected during the first waves of the HIV epidemic in China via blood donation and drug injection, while more recently diagnosed patients were infected through sexual contact. Also, the increased knowledge of HIV/AIDS and access to ART may have contributed to a decrease in the mental burden of PLWH. The current study shows that the prevalence rate of depression among the MSM population was 34%, consistent with the result of 36% from a recent study (43).

We found that the internal reliability of the HADS was satisfactory, which is similar to previous findings using the Chinese version of the test (27, 28, 31) and in PLWH (26). A moderate correlation (<0.80) between the anxiety and depression subscales was also found, suggesting an acceptable discriminant validity (44).

Item 7, which is an original anxiety-subscale item, was found to have significant loading onto the depression factor, but it was below the cutoff point for the anxiety factor in this study. Anomalous loading of item 7 has been frequently reported in previous studies, including assays using the Chinese version of the HADS (24, 28, 30, 45) and in PLWH (46) and, thus, was considered a poor item (25). Some authors argued that item 7 (“I can sit at ease and feel relaxed”) loads on both subscales because it refers to psychomotor agitation “cannot sit at ease” and to the inner tension or anhedonia domain “cannot feel relaxed” of depression (18, 47). Another reason that possibly explains the anomalous loading of item 7 is that it has a response that is opposite to that of item 6, which precedes it, thus disorienting patients and making them ignore the change in arrangement of the items and scoring unless they are particularly vigilant (19). The HADS contains six reversed wording items, of which five items belong to the anxiety subscale (items 2, 4, 6, 12, and 14) and only one item (item 7) belongs to the depression subscale. The unequal distribution of reversed wording items is thought to be the cause of anomalous loadings (19) as negative wording items may have more influence on vulnerable populations, such as people with lower levels of schooling (48, 49). These results indicate that the item-wording effects of the HADS should be taken into consideration if applied to vulnerable populations, such as PLWH in underdeveloped areas. Furthermore, the addition of an item-wording factor that contained six reversed wording items and no correlation with other factors improved the model fit (20, 49), which supported a tripartite-like structure and thus supported the existence of a general factor (50). Rescoring of some of the items based on the IRT may improve the utility of the HADS (45).

We also found that item 8 on the depression subscale had an anomalous loading, loading significantly onto the anxiety subscale instead of on the depression subscale. Anomalous loadings of item 8 were also frequently reported (15, 25, 51), including in PLWH (46). One possible interpretation is that “slowed down” may have been misinterpreted as a somatic illness or cognitive slowness instead of the intended depressive symptom of fatigue (52). Furthermore, in the Chinese version of item 8, “slowed down” is translated more like “dullness,” which may lead to more misunderstanding. Anomalous loading of item 8 was found in the elderly (15), and thus, item 8 was thought to be an age-related slow down (53). But considering the average age of 37.6 in the current study, this may not be the case. The HADS contains colloquial British expressions, such as “butterflies in the stomach” in item 9, which causes trouble and inaccuracy when translating into other languages (i.e., “trembling with fear” in the Chinese version); however, it did not show anomalous loadings in the present study. Some authors have argued that little attention has been paid to the translated version of the HADS used in the study, despite the fact that non-English-speaking countries have carried out such studies more often and that the translation of the HADS may explain the inconsistencies in factor structure analyses (54). Cultural factors may also influence respondents’ comprehension of the HADS, such as among HIV-positive sex workers in Bengali; “slowed down” is possibly interpreted as succumbing to HIV (46). Educational level may also influence the responses for some items for reasons other than the wording effect (45). Also, the ability of an item (i.e., item 11: “I feel restless as if I have to be on the move”) to distinguish psychopathology from normal individual differences in personality declines as it intends to be inexplicit when referring to psychiatric symptoms to avoid any stigma (19). Thus, translated versions of the HADS need to be revised according to the language and culture of the specific population to facilitate cross-cultural comparison.

Another depression subscale item with anomalous loading is item 10, which loaded “fairly” onto the anxiety subscale and did not reach the cutoff for loading onto the depression subscale. Although item 10 is not such a poor item, anomalous loading of item 10 can also be frequently found (55, 56, 57), including in the Chinese version of the HADS (28) and in a sample of PLWH (49). Item 10 was reported to have an age-related bias (58) and was thought to be influenced by interpersonal attraction or social desirability (47), making anomalies understandable considering the stigma present in the sample of PLWH aged 18 to 81 in the current study. The HADS-D subscale is problematic because it focuses almost entirely on anhedonia and ignored somatic symptoms and other important factors (e.g., feelings of worthlessness or guilt; difficulty concentrating, indecisiveness) that are currently included in the diagnostic criteria for depression (19, 59). The depression subscale only includes 4 out of 13 diagnostic symptoms of major depressive disorder (60). There is limited evidence showing that somatic symptoms in medical patients are less valid indicators of depression (19) and that medical patients score higher than psychiatric patients on conventional somatic items assessed by other measures, matched for age and cognitive/affective items (61, 62), which runs contrary to the theory on which the HADS was designed (7). Furthermore, some studies have shown that somatic–cognitive symptoms appear to be distinct components of depression in PLWH (63, 64), especially in non-Western settings, where depression is experienced more somatically (65). Some antiviral drugs, such as efavirenz, cause significant neuropsychiatric complications, including depression (66), which makes measuring depression in PLWH more complex. HIV-associated depression commonly shows symptoms of insomnia, loss of appetite, anhedonia, and difficulties with memory and concentration (67), suggesting that a combination of sleep and appetite disturbances may be more revealing of depression among medical patients than item 10.

The latent structure of the HADS has long been debated, but recent evidence points to a bifactor model (20). Moreover, the EFA studies of the HADS typically found most items loading highly on the first unrotated factor (68), and several psychometric methods, including EFA, show only one level of a hierarchical dimensionality structure, and multidimensionality is an inclination of researchers (57). Also, most IRT studies found one-dimensional solutions (25), indicating the presence of a general factor. The bifactor model and Dunbar’s high-order model were considered understandable solutions in the presence of a strong general factor (20), but the former was considered to be a better hierarchical explanation (20) and proved to be superior in subsequent studies (21–23). Furthermore, it is difficult to distinguish anxiety from depression as they are commonly comorbid and have overlapping symptoms (69). Also, factor analyses showed that anxiety and depression subscales often yield highly correlated assessments (15, 56, 70) and that the correlation was indispensable even in a bifactor model (21).

Considering the presence of a strong general factor and the indistinguishable relationship between anxiety and depression, using the HADS as a measurement of general distress instead of anxiety or depression separately was recommended prematurely. Further studies have revealed that the HADS anxiety subscale has equal ability as a depression subscale in screening for depressive disorders (61). The HADS’ total scale outperformed anxiety and depression subscales in screening for anxiety and depression disorders, respectively (61, 71), indicating the suitability of using the HADS as a total scale. Studies on the case-finding ability of the HADS confirmed the practical value of the HADS’ total scale in identification of “emotional distress” (72). However, caution should be exercised when using the HADS’ total scale to evaluate general distress because relevant psychological symptoms other than anxiety or depression are excluded from the scale. More recent studies testing the psychometric properties of the HADS’ total scale showed good sensitivity and specificity, superior to other scales (73), with a cutoff score of 11/12 (12), 14/15 (73), 15/16 (74), or 16/17 (73) in different populations. Yet, the psychometric properties of the HADS’ total scale need to be further studied.

Some authors found that the HADS shows high sensitivity to reflect changes resulting from treatment (75) and thus can be used to measure the improvement of mental status after medical treatment. An IRT study revealed that some items were difficult and could detect severe anxiety and/or depression (76). These provide new aspects for the practical application of the HADS other than for measuring general distress.

The HADS holds measurement invariance between men and women, which supports earlier studies using the Chinese version (27, 28), and between MSM and non-MSM, while no former study has tested the measurement invariance between courses of infection in HIV-positive populations. The finding that the HADS works similarly across different courses of infection facilitates the use of the HADS in clinical practice as MSM patients may dissemble their sexual identity because of the stigma.

## Strengths and Limitations

The current study provides a significant contribution to the existing literature as it is the first study to analyze the factor structures of the HADS in PLWH in China. Additionally, the sample size of 4,102 is larger than most other studies at present. A sophisticated set of analyses was performed, including measurement of invariance, which allows for direct testing of model fit across different groups of patients. Also, the current study tested a bifactor model and found it to be the best fit model, which supports the recent theory of using the HADS as a general scale. Several limitations need to be taken into account when interpreting the findings of the current study. First, the absence of a gold standard diagnostic measure for anxiety or depression made the analysis of sensitivity and specificity impossible. Therefore, the external validity was unable to be analyzed due to the lack of an external criterion. Additionally, the cutoff score could not be calculated in this sample without a gold standard. Second, IRT studies should be conducted to discover whether rescoring or revising of the HADS is necessary. Third, the lack of data from a noninfected control made the comparison of mental status between these two groups impossible.

## Conclusion

The current study presents a psychometric assessment of the Chinese version of the HADS in a large sample of PLWH. The HADS can be used as a total scale that measures general psychological distress, instead of anxiety and depression separately, when applied to PLWH. A valid scale, such as the HADS, can help improve the mental health of PLWH in China and, thus, assist in their treatment and improve their quality of life.

## Author Contributions

Formal analysis was done by ZY and JH. Data curation was done by AS and TZ. Original draft preparation was performed by ZY and XH. Review and editing were handled by KM, WW and HC. Supervision was done by HC and HW. Project administration was handled by XL.

## Conflict of Interest Statement

The authors declare that the research was conducted in the absence of any commercial or financial relationships that could be construed as a potential conflict of interest.

## References

[1] WHO (2018). HIV/AIDS, Retrieved from http://www.who.int/news-room/fact-sheets/detail/hiv-aids.

[2] Lowther K Selman L Harding R Higginson IJ Experience of persistent psychological symptoms and perceived stigma among people with HIV on antiretroviral therapy (ART): a systematic review. Int J Nurs Stud (2014) 51(8):1171–89. 10.1016/j.ijnurstu.2014.01.015 24602830

[3] Niu L Luo D Liu Y Silenzio VM Xiao S The mental health of people living with HIV in China, 1998-2014: a systematic review. PloS One (2016) 11(4):e0153489. 10.1371/journal.pone.0153489 27082749 PMC4833336

[4] Mayston R Kinyanda E Chishinga N Prince M Patel V Mental disorder and the outcome of HIV/AIDS in low-income and middle-income countries: a systematic review. AIDS (2012) 26 Suppl 2:S117–135. 10.1097/QAD.0b013e32835bde0f 23303434

[5] Tao J Vermund SH Qian HZ Association between depression and antiretroviral therapy use among people living with HIV: a meta-analysis. AIDS Behav (2018) 22(5):1542–50. 10.1007/s10461-017-1776-8 PMC7942230 28439754

[6] Orza L Bewley S Logie CH Crone ET Moroz S Strachan S How does living with HIV impact on women’s mental health? Voices from a global survey. J Int AIDS Soc (2015) 18(Suppl 5):20289. 10.7448/IAS.18.6.20289 26643460 PMC4672402

[7] Zigmond AS Snaith RP The Hospital Anxiety and Depression Scale. Acta Psychiatr Scand (1983) 67(6):361–70. 10.1111/j.1600-0447.1983.tb09716.x 6880820

[8] Savard J Laberge B Gauthier JG Ivers H Bergeron MG Evaluating anxiety and depression in HIV-infected patients. J Pers Assess (1998) 71(3):349–67. 10.1207/s15327752jpa7103_5 9933941

[9] Bjelland I Dahl AA Haug TT Neckelmann D The validity of the Hospital Anxiety and Depression Scale. J Psychosom Res (2002) 52(2):69–77. 10.1016/S0022-3999(01)00296-3 11832252

[10] Yamamoto-Furusho JK Sarmiento-Aguilar A Garcia-Alanis M Gomez-Garcia LE Toledo-Maurino J Olivares-Guzman L Hospital Anxiety and Depression Scale (HADS): validation in Mexican patients with inflammatory bowel disease. Gastroenterol Hepatol (2018) 41(8):477–82. 10.1016/j.gastrohep.2018.05.009 29937084

[11] Villoria E Lara L Assessment of the Hospital Anxiety and Depression Scale for cancer patients. Revista Medica De Chile (2018) 146(3):300–7. 10.4067/s0034-98872018000300300 29999099

[12] Miljanovic M Sindik J Milunovic V Skoc VK Bras M Dordevic V Factor structure and cut-off scores of the Hospital Anxiety and Depression scale (HADS) in a Croatian sample of adult patients suffering from advanced cancer. Psychiatr Danub (2017) 29(4):451–8. 10.24869/psyd.2017.451 29197202

[13] Terkawi AS Tsang S AlKahtani GJ Al-Mousa SH Al Musaed S AlZoraigi US Development and validation of Arabic version of the Hospital Anxiety and Depression Scale. Saudi J Anaesth (2017) 11(5):S11–S18. 10.4103/sja.SJA_43_17 28616000 PMC5463562

[14] Lin C-Y Pakpour AH Using Hospital Anxiety and Depression Scale (HADS) on patients with epilepsy: confirmatory factor analysis and Rasch models. Seizure-European J Epilepsy (2017) 45:42–6. 10.1016/j.seizure.2016.11.019 27915110

[15] Djukanovic I Carlsson J Arestedt K Is the Hospital Anxiety and Depression Scale (HADS) a valid measure in a general population 65–80 years old? A psychometric evaluation study. Health Qual Life Outcomes (2017) 15(1):193. 10.1186/s12955-017-0759-9 28978356 PMC5628437

[16] Wiglusz MS Landowski J Michalak L Cubala WJ Validation of the Hospital Anxiety and Depression Scale in patients with epilepsy. Epilepsy & Behavior (2016) 58:97–101. 10.1016/j.yebeh.2016.03.003 27064829

[17] Phan T Carter O Adams C Waterer G Chung LP Hawkins M Discriminant validity of the Hospital Anxiety and Depression Scale, Beck Depression Inventory (II) and Beck Anxiety Inventory to confirmed clinical diagnosis of depression and anxiety in patients with chronic obstructive pulmonary disease. Chron Respir Dis (2016) 13(3):220–8. 10.1177/1479972316634604 PMC5720182 26944070

[18] Dunbar M Ford G Hunt K Der G A confirmatory factor analysis of the Hospital Anxiety and Depression Scale: comparing empirically and theoretically derived structures. Br J Clin Psychol (2000) 39(Pt 1):79–94. 10.1348/014466500163121 10789030

[19] Coyne JC van Sonderen E No further research needed: abandoning the Hospital and Anxiety Depression Scale (HADS). J Psychosom Res (2012) 72(3):173–4. 10.1016/j.jpsychores.2011.12.003 22325694

[20] Norton S Cosco T Doyle F Done J Sacker A The Hospital Anxiety and Depression Scale: a meta confirmatory factor analysis. J Psychosom Res (2013) 74(1):74–81. 10.1016/j.jpsychores.2012.10.010 23272992

[21] Burns A Hofer S Curry P Sexton E Doyle F Revisiting the dimensionality of the Hospital Anxiety and Depression Scale in an international sample of patients with ischaemic heart disease. J Psychosom Res (2014) 77(2):116–21. 10.1016/j.jpsychores.2014.05.005 25077852

[22] Iani L Lauriola M Costantini M A confirmatory bifactor analysis of the Hospital Anxiety and Depression Scale in an Italian community sample. Health Qual Life Outcomes (2014) 12:84. 10.1186/1477-7525-12-84 24902622 PMC4054905

[23] Dale M Maltby J Martucci R Shimozaki S, REGISTRY investigators of the European Huntington’s Disease Network Factor analysis of the Hospital Anxiety and Depression Scale among a Huntington’s disease population. Mov Disord (2015) 30(14):1954–60. 10.1002/mds.26419 26443751

[24] Lee CP Chou YH Liu CY Hung CI Dimensionality of the Chinese Hospital Anxiety Depression Scale in psychiatric outpatients: mokken scale and factor analyses. Int J Psychiatry Clin Pract (2017) 21(4):283–91. 10.1080/13651501.2017.1311350 28417655

[25] Cosco TD Doyle F Ward M McGee H Latent structure of the Hospital Anxiety And Depression Scale: a 10-year systematic review. J Psychosom Res (2012) 72(3):180–4. 10.1016/j.jpsychores.2011.06.008 22325696

[26] Reda AA Reliability and validity of the Ethiopian version of the Hospital Anxiety and Depression Scale (HADS) in HIV infected patients. Plos One (2011) 6(1):e16049. 10.1371/journal.pone.0016049 21283565 PMC3026786

[27] Fong TC Ho RT Testing gender invariance of the hospital anxiety and depression scale using the classical approach and Bayesian approach. Qual Life Res (2014) 23(5):1421–6. 10.1007/s11136-013-0594-3 24307211

[28] Chan YF Leung DY Fong DY Leung CM Lee AM Psychometric evaluation of the Hospital Anxiety and Depression Scale in a large community sample of adolescents in Hong Kong. Qual Life Res (2010) 19(6):865–73. 10.1007/s11136-010-9645-1 PMC2892613 20373037

[29] Li Q Lin Y Hu C Xu Y Zhou H Yang L The Chinese version of Hospital Anxiety and Depression Scale: psychometric properties in Chinese cancer patients and their family caregivers. Eur J Oncol Nurs (2016) 25:16–23. 10.1016/j.ejon.2016.09.004 27865248

[30] Wang W Lopez V Martin CR Structural ambiguity of the Chinese version of the Hospital Anxiety and Depression Scale in patients with coronary heart disease. Health Qual Life Outcomes (2006) 4:6. 10.1186/1477-7525-4-6 16438711 PMC1360658

[31] Martin CR Thompson DR Chan DS An examination of the psychometric properties of the Hospital Anxiety and Depression Scale in Chinese patients with acute coronary syndrome. Psychiatry Res (2004) 129(3):279–88. 10.1016/j.psychres.2004.06.012 15661322

[32] Wu X Lau JTF Mak WWS Gu J Mo PKH Wang X How newly diagnosed HIV-positive men who have sex with men look at HIV/AIDS—validation of the Chinese version of the revised illness perception questionnaire. BMC Infect Dis (2018) 18(1):2. 10.1186/s12879-017-2902-y 29291733 PMC5748952

[33] Murphy PJ Garrido-Hernansaiz H Mulcahy F Hevey D HIV-related stigma and optimism as predictors of anxiety and depression among HIV-positive men who have sex with men in the United Kingdom and Ireland. AIDS Care (2018) 30(9):1173–9. 10.1080/09540121.2018.1445827 29494229

[34] Tabachnick BG Fidell LS Using multivariate statistics. 5th ed Boston, MA: Allyn and Bacon (2007).

[35] Brown TA Confirmatory factor analysis for applied research. New York: Guilford Publications (2015).

[36] Chen FF Sensitivity of goodness of fit indexes to lack of measurement invariance. Struct Eqn Model (2007) 14:464–504. 10.1080/10705510701301834

[37] Widaman KF Reise SP Exploring the measurement invariance of psychological instruments: applications in the substance use domain. Ariel (1997) 165:220–14. 10.1037/10222-009

[38] Kline P The Handbook of Psychological Testing. London: Routledge (1999).

[39] Mykletun A Stordal E Dahl AA Hospital Anxiety and Depression Scale (HAD) scale: factor structure, item analyses and internal consistency in a large population. Br J Psychiatry (2001) 179:540–4. 10.1192/bjp.179.6.540 11731359

[40] Buysse DJ Reynolds CF Monk TH Berman SR Kupfer DJ The Pittsburgh Sleep Quality Index: a new instrument for psychiatric practice and research. Psychiatry Res (1989) 28(2):193–213. 10.1016/0165-1781(89)90047-4 2748771

[41] Darko DF McCutchan JA Kripke DF Gillin JC Golshan S Fatigue, sleep disturbance, disability, and indices of progression of HIV infection. Am J Psychiatry (1992) 149(4):514–20. 10.1176/ajp.149.4.514 1554037

[42] Di Riso D Bobbio A Chessa D Lis A Mazzeschi C Analysis of the interplay between depression, anxiety, and psychological resources in adolescence using self-report measures. Int J Psychiatry Clin Pract (2014) 18(2):103–11. 10.3109/13651501.2014.890227 24494776

[43] Tao J Vermund SH Lu H Ruan Y Shepherd BE Kipp AM Impact of depression and anxiety on initiation of antiretroviral therapy among men who have sex with men with newly diagnosed HIV infections in China. AIDS Patient Care STDS (2017) 31(2):96–104. 10.1089/apc.2016.0214 28170305 PMC5312604

[44] Farrell AM Insufficient discriminant validity: a comment on Bove, Pervan, Beatty, and Shiu (2009). J Bus Res (2010) 63:324–7. 10.1016/j.jbusres.2009.05.003

[45] Lin X Chen Z Jin L Gao W Qu B Zuo Y Rasch analysis of the Hospital Anxiety and Depression Scale among Chinese cataract patients. PloS One (2017) 12(9):e0185287. 10.1371/journal.pone.0185287 28949992 PMC5614566

[46] Ghose T Chowdhury A Solomon P Ali S Depression and anxiety among HIV-positive sex workers in Kolkata, India: testing and modifying the Hospital Anxiety Depression Scale. Int Soc Work (2015) 58(2):211–22. 10.1177/0020872813497381

[47] Matsudaira T Igarashi H Kikuchi H Kano R Mitoma H Ohuchi K Factor structure of the Hospital Anxiety and Depression Scale in Japanese psychiatric outpatient and student populations. Health Qual Life Outcomes (2009) 7:42. 10.1186/1477-7525-7-42 19445722 PMC2687424

[48] Schmitt DP Allik J Simultaneous administration of the Rosenberg Self-Esteem Scale in 53 nations: exploring the universal and culture-specific features of global self-esteem. J Pers Soc Psychol (2005) 89(4):623–42. 10.1037/0022-3514.89.4.623 16287423

[49] Wouters E Booysen Fle R Ponnet K Baron Van Loon F Wording effects and the factor structure of the Hospital Anxiety & Depression Scale in HIV/AIDS patients on antiretroviral treatment in South Africa. PloS One (2012) 7(4):e34881. 10.1371/journal.pone.0034881 22536338 PMC3335020

[50] Norton S Sacker A Done J Further research needed: a comment on Coyne and van Sonderen’s call to abandon the Hospital Anxiety and Depression Scale. J Psychosom Res (2012) 73(1):75–76. 10.1016/j.jpsychores.2012.04.005 22691565

[51] Saez-Flores E Tonarely NA Barker DH Quittner AL Examining the stability of the Hospital Anxiety and Depression Scale factor structure in adolescents and young adults with cystic fibrosis: a confirmatory factor analysis. J Pediatr Psychol (2018) 43(6):625–35. 10.1093/jpepsy/jsx155 29309626

[52] Johnston M Pollard B Hennessey P Construct validation of the Hospital Anxiety and Depression Scale with clinical populations. J Psychosom Res (2000) 48(6):579–84. 10.1016/S0022-3999(00)00102-1 11033377

[53] Helvik AS Engedal K Skancke RH Selbaek G A psychometric evaluation of the Hospital Anxiety and Depression Scale for the medically hospitalized elderly. Nord J Psychiatry (2011) 65(5):338–44. 10.3109/08039488.2011.560684 21341979

[54] Maters GA Sanderman R Kim AY Coyne JC Problems in cross-cultural use of the Hospital Anxiety and Depression Scale: “No butterflies in the desert”. PloS One (2013) 8(8):e70975. 10.1371/journal.pone.0070975 23976969 PMC3743400

[55] Emons WH Sijtsma K Pedersen SS Dimensionality of the Hospital Anxiety and Depression Scale (HADS) in cardiac patients: comparison of Mokken scale analysis and factor analysis. Assessment (2012) 19(3):337–53. 10.1177/1073191110384951 20947706

[56] Haugan G Drageset J The Hospital Anxiety and Depression Scale—dimensionality, reliability and construct validity among cognitively intact nursing home patients. J Affect Disord (2014) 165:8–15. 10.1016/j.jad.2014.04.042 24882171

[57] Straat JH van der Ark LA Sijtsma K Methodological artifacts in dimensionality assessment of the Hospital Anxiety and Depression Scale (HADS). J Psychosom Res (2013) 74(2):116–21. 10.1016/j.jpsychores.2012.11.012 23332525

[58] Verdam MGE Oort FJ Sprangers MAG Item bias detection in the Hospital Anxiety and Depression Scale using structural equation modeling: comparison with other item bias detection methods. Qual Life Res (2017) 26(6):1439–50. 10.1007/s11136-016-1469-1 PMC5420371 27943018

[59] Snaith RP The Hospital Anxiety and Depression Scale. Health Qual Life Outcomes (2003) 1:29. 10.1186/1477-7525-1-29 12914662 PMC183845

[60] Doyle F Conroy R McGee H Challenges in reducing depression-related mortality in cardiac populations: cognition, emotion, fatigue or personality? Health Psychol Rev (2017) 1:137–72. 10.1080/17437190802046322

[61] Mitchell AJ Meader N Symonds P Diagnostic validity of the Hospital Anxiety and Depression Scale (HADS) in cancer and palliative settings: a meta-analysis. J Affect Disord (2010) 126(3):335–48. 10.1016/j.jad.2010.01.067 20207007

[62] Thombs BD Ziegelstein RC Pilote L Dozois DJ Beck AT Dobson KS Somatic symptom overlap in Beck Depression Inventory-II scores following myocardial infarction. Br J Psychiatry (2010) 197(1):61–6. 10.1192/bjp.bp.109.076596 PMC2894982 20592436

[63] Ashaba S Kakuhikire B Vorechovska D Perkins JM Cooper-Vince CE Maling S Reliability, validity, and factor structure of the Hopkins Symptom Checklist-25: population-based study of persons living with HIV in rural Uganda. AIDS Behav (2018) 22(5):1467–74. 10.1007/s10461-017-1843-1 PMC6613367 28667469

[64] Psaros C Haberer JE Boum Y Tsai AC Martin JN Hunt PW The factor structure and presentation of depression among HIV-positive adults in Uganda. AIDS Behav (2015) 19(1):27–33. 10.1007/s10461-014-0796-x 24854877 PMC4360967

[65] Okello ES Neema S Explanatory models and help-seeking behavior: pathways to psychiatric care among patients admitted for depression in Mulago hospital, Kampala, Uganda. Qual Health Res (2007) 17(1):14–25. 10.1177/1049732306296433 17170240

[66] Treisman GJ Soudry O Neuropsychiatric effects of HIV antiviral medications. Drug Saf (2016) 39(10):945–57. 10.1007/s40264-016-0440-y 27534750

[67] Wolff LC Alvarado MR Wolff RM [Depression in HIV infection: prevalence, risk factors and management]. Rev Chilena Infectol (2010) 27(1):65–74. 10.4067/S0716-10182010000100011 20140318

[68] Herrmann C International experiences with the Hospital Anxiety and Depression Scale—A review of validation data and clinical results. J Psychosom Res (1997) 42(1):17–41. 10.1016/S0022-3999(96)00216-4 9055211

[69] Watson D Rethinking the mood and anxiety disorders: a quantitative hierarchical model for DSM-V. J Abnorm Psychol (2005) 114(4):522–36. 10.1037/0021-843X.114.4.522 16351375

[70] Boxley L Flaherty JM Spencer RJ Drag LL Pangilinan PH Bieliauskas LA Reliability and factor structure of the Hospital Anxiety and Depression Scale in a polytrauma clinic. J Rehabil Res Dev (2016) 53(6):873–80. 10.1682/JRRD.2015.05.0088 28273327

[71] Chan CYY Tsang HHL Lau CS Chung HY Prevalence of depressive and anxiety disorders and validation of the Hospital Anxiety and Depression Scale as a screening tool in axial spondyloarthritis patients. Int J Rheum Dis (2017) 20(3):317–25. 10.1111/1756-185X.12456 25293872

[72] Brennan C Worrall-Davies A McMillan D Gilbody S House A The Hospital Anxiety and Depression Scale: a diagnostic meta-analysis of case-finding ability. J Psychosom Res (2010) 69(4):371–8. 10.1016/j.jpsychores.2010.04.006 20846538

[73] Schellekens MPJ van den Hurk DGM Prins JB Molema J van der Drift MA Speckens AEM The suitability of the Hospital Anxiety and Depression Scale, Distress Thermometer and other instruments to screen for psychiatric disorders in both lung cancer patients and their partners. J Affect Disord (2016) 203:176–83. 10.1016/j.jad.2016.05.044 27295374

[74] Roberge P Dore I Menear M Chartrand E Ciampi A Duhoux A A psychometric evaluation of the French Canadian version of the Hospital Anxiety and Depression Scale in a large primary care population. J Affect Disord (2013) 147(1–3):171–9. 10.1016/j.jad.2012.10.029 23218249

[75] Turk DC Dworkin RH Trudeau JJ Benson C Biondi DM Katz NP Validation of the Hospital Anxiety and Depression Scale in patients with acute low back pain. J Pain (2015) 16(10):1012–21. 10.1016/j.jpain.2015.07.001 26208762

[76] Ayis SA Ayerbe L Ashworth M Wolfe C Evaluation of the Hospital Anxiety and Depression Scale (HADS) in screening stroke patients for symptoms: item Response Theory (IRT) analysis. J Affect Disord (2018) 228:33–40. 10.1016/j.jad.2017.11.037 29202444

